# Nicotine enhances mesangial cell proliferation and fibronectin production in high glucose milieu via activation of Wnt/β-catenin pathway

**DOI:** 10.1042/BSR20180100

**Published:** 2018-05-31

**Authors:** Xiqian Lan, Hongxiu Wen, Rukhsana Aslam, Seyedeh Shadafarin Marashi Shoshtari, Abheepsa Mishra, Vinod Kumar, Haichao Wang, Guisheng Wu, Huairong Luo, Ashwani Malhotra, Pravin C. Singhal

**Affiliations:** 1 Key Laboratory for Aging and Regenerative Medicine, School of Pharmacy, Southwest Medical University, Luzhou, Sichuan, China; 2 Feinstein Institute for Medical Research and Hofstra NorthWell Medical School, Manhasset, NY, U.S.A.; 3 Department of Emergency Medicine, North Shore University Hospital, Manhasset, NY, U.S.A.

**Keywords:** nicotine, high glucose, mesangial cell, proliferation, Wnt/β-catenin pathway

## Abstract

Diabetic nephropathy (DN) is a major complication of diabetes mellitus. Clinic reports indicate cigarette smoking is an independent risk factor for chronic kidney disease including DN; however, the underlying molecular mechanisms are not clear. Recent studies have demonstrated that nicotine, one of the active compounds in cigarette smoke, contributes to the pathogenesis of the cigarette smoking-accelerated chronic kidney disease. One of the characteristics of DN is the expansion of mesangium, a precursor of glomerular sclerosis. In the present study, we examined the involvement of Wnt/β-catenin pathway in nicotine-mediated mesangial cell growth in high glucose milieu. Primary human renal mesangial cells were treated with nicotine in the presence of normal (5 mM) or high glucose (30 mM) followed by evaluation for cell growth. In the presence of normal glucose, nicotine increased both the total cell numbers and Ki-67 positive cell ratio, indicating that nicotine stimulated mesangial cell proliferation. Although high glucose itself also stimulated mesangial cell proliferation, nicotine further enhanced the mitogenic effect of high glucose. Similarly, nicotine increased the expression of Wnts, β-catenin, and fibronectin in normal glucose medium, but further increased mesangial cell expression of these proteins in high glucose milieu. Pharmacological inhibition or genetic knockdown of β-catenin activity or expression with specific inhibitor FH535 or siRNA significantly impaired the nicotine/glucose-stimulated cell proliferation and fibronectin production. We conclude that nicotine may enhance renal mesangial cell proliferation and fibronectin production under high glucose milieus partly through activating Wnt/β-catenin pathway. Our study provides insight into molecular mechanisms involved in DN.

## Introduction

Diabetic nephropathy (DN) is a chronic kidney disease that manifests in the form of progressive proteinuria, decline in glomerular filtration rate (GFR), hypertension, and a higher risk of cardiovascular morbidity and mortality. Kidney pathology is characterized by thickening of the glomerular and tubular basement membrane, mesangial expansion, and tubulo-interstitial fibrosis. As of 2017, an estimated 425 million people (1 in 11 adults) had diabetes worldwide, and 12% of global health expenditure is spent on diabetes ($727 billion) [1]. In U.S.A. and China, the estimated diabetes people were 30.3 and 110 million, respectively [2,3]. Amongst the causes of end-stage kidney disease (ESKD) in the world, diabetes mellitus accounted for 30–47% [4]. Since causes and molecular mechanisms of DN are still not clear, therefore, it is difficult to prevent and treat; moreover, the existing management of diabetic kidney patients constitutes a tremendous socioeconomic burden on society [1]. Therefore, improving mechanistic understanding of DN and developing new and effective therapeutic approaches to prevent or delay the progression of DN are needed.

Cigarette smoking is amongst the top ten contributors of morbidity and mortality of human population [5]. Increasing evidences have demonstrated that cigarette smoking is an independent risk factor for the development and progression of chronic kidney disease including DN [6–10]. Recent studies have demonstrated that nicotine, one of the highly active compounds in cigarette smoke, contributes to the pathogenesis of smoking-mediated kidney dysfunction [11,12]. Nicotine plays its role through activation of specific nicotinic ACh receptors (nAChRs). Studies from our group and others have confirmed that the nAChRs are expressed in kidney cells including podocytes, tubular epithelial cells, and mesangial cells. In our previous studies, we found that stimulating podocyte, one of the most important epithelial cells for blood–urine barrier, activates MAPK kinases and enhances endogenous ROS generation, which promotes podocyte apoptosis [13]. It is reported that short-term stimulation of tubular epithelial cells with high dose of nicotine (200–400 μM) causes apoptosis or epithelial–mesenchymal transition (EMT) through enhanced ROS generation [14,15]; while long-term stimulation with low dose of nicotine (1–10 μM) increases proliferation through activaton of AKT [16]. For mesangial cell, nicotine stimulation activates TGF-β pathway, which promotes the cell proliferation and production of extracellular matrix proteins [17–19]. These studies show that nicotine stimulation causes injuries to the kidney cells, leading to renal dysfunction. However, the molecular mechanisms underlying nicotine-accelerated DN have not been well elucidated yet.

Mesangial cells are specialized cells that reside in the glomerular mesangium along with the mesangial matrix in the center of capillary loops. They play an important role in the maintenance of glomerular filtration, and respond to local injury in the form of proliferation, apoptosis, and matrix remodeling [20]. Mesangial cell proliferation is associated with extracellular matrix accumulation, while a decrease in proliferation is associated with an attenuated mesangial sclerosis [21–23]. Previous reports have shown that nicotine promotes mesangial cell proliferation and fibronectin production through activation of TGF-β signaling pathway [17–19], but its role on mesangial cell growth in high glucose milieu has not been investigated. In addition, the pleiotropy of TGF-β makes it not suitable for therapeutic target. We hypothesized that nicotine enhances mesangial cell proliferation and fibronectin production in high glucose milieu through Wnt/β -catenin pathway.

## Materials and methods

### Culture of mesangial cell

Primary human renal mesangial cells were purchased from ScienCell (Carlsbad, CA), grown in Mesangial Cell Medium (ScienCell) supplemented with 2% FBS and 1% mesangial cell growth supplement (ScienCell). Cells were passaged by trypsinization when confluent and used between the third and ninth passages. Before treatment, cells were cultured in serum-free RPMI medium with 5 mM glucose for 24 h.

### Real-time PCR

Total RNA was isolated from human renal mesangial cells using TRIzol reagent (Invitrogen). Five micrograms of total RNA were reverse transcribed using the first-strand synthesis system (Invitrogen). Real time-PCR was performed in a Prism 7900HT sequence-detection system (Applied Biosystems, Foster City, CA, U.S.A.). Relative mRNA levels were determined and standardized with a GAPDH internal control using comparative ΔΔ*C*_T_ method. GAPDH was used as an internal control. Sequences of primers are listed in Table 1.

**Table 1:** Primer sequences for real-time PCR

Gene	Forward primer	Reverse primer
*Wnt2*	TAGTCGGGAATCTGCCTTTG	TTCCTTTCCTTTGCATCCAC
*Wnt2B*	CTCATCAGCAGGGGTAGTCC	AAAACGGACACCGTAGTGGA
*Wnt3*	ACGAGAACTCCCCCAACTTT	GATGCAGTGGCATTTTTCCT
*Wnt6*	GGTTATGGACCCTACCAGCA	AATGTCCTGTTGCAGGATGC
*Wnt7B*	GCAAGTGGATTTTCTACGTG	TGACAGTGCTCCGAGCTTCA
*β-catenin*	GCAGAAAATGGTTGCCTTGCTC	AGCACCTTCAGCACTCTGCTTG
*GAPDH*	CCATGGAGAAGGCTGGGC	CAAAGTTGTCATGGATGA

### Western blotting analysis

Western blotting was performed using established methodology [13]. Briefly, cells were washed with PBS and lysed in RIPA buffer. Proteins (20–30 μg) were separated by 10–12% SDS/PAGE and then transferred on an immunoblot PVDF membrane (Bio-Rad, Hercules, CA). After blocking in PBS/Tween (0.1%) with 5% nonfat milk, the membrane was incubated with primary antibodies overnight at 4°C followed by horseradish peroxidase-conjugated secondary antibodies (Santa Cruz Technology, sc-2033 for anti-goat, and sc-2004 for anti-rabbit, 1:3000) and then developed using ECL solution (Pierce). Primary antibodies used were rabbit anti-β-catenin (Abcam, ab32572, 1:1000), rabbit anti-fibronectin (Santa Cruz Technology, sc-9068, 1:1000), rabbit anti-Wnt3 (Abcam, ab32249, 1:1000), rabbit anti-Wnt7B (Abcam, ab94915, 1:1000), and goat anti-actin (Santa Cruz Technology, sc-1616, 1:3000). For protein expression quantitation, the films were scanned with a CanonScan 9950F scanner and the acquired images were then analyzed using the public domain NIH image program (http://rsb.info.nih.gov/nih-image/).

### Ki-67 staining

Mesangial cells (5 × 10^4^) were planted in 35-mm dishes, and after appropriate treatment, immunofluorescent staining was performed as reported previously [13]. Briefly, the medium was removed, and the cells were successively fixed with 4% PFA, permeabilized with 0.3% Triton X-100, and were blocked with 2% BSA in 0.1% Triton X-100. Then, the cells were incubated with primary antibody, rabbit anti-Ki-67 (Santa Cruz Technology, sc-15402, 1:100), overnight at 4°C, followed by Alexa Fluor secondary antibodies (Invitrogen, A10042, 1:800), donkey anti-rabbit IgG Alexa Fluor 568 for 1 h at room temperature. Nuclei were stained with Hoechest 33342. Staining results were visualized and captured with a ZEISS microscope, and Ki-67 positive cells were counted.

### ELISA

Wnt3 in the medium of mesangial cell was measured by using the WNT3 ELISA Kit (Cat# OKCD00611, Aviva Systems Biology, San Diego, CA), following the manufacturer’s instructions.

### Statistical analyses

Data were presented as means ± S.D. unless otherwise noted. All experiments were repeated at least three times with duplicate or triplicate samples in each assay. All data were evaluated statistically by ANOVA, followed by Newman–Keuls multiple comparison tests using software (Prism 4.0, GraphPad Software). In the case of single mean comparison, data were analyzed by *t* test. *P*-values <0.05 were regarded as statistically significant.

## Results and discussion

### Nicotine enhances mesangial cell proliferation in high glucose milieu

**Figure 1:**
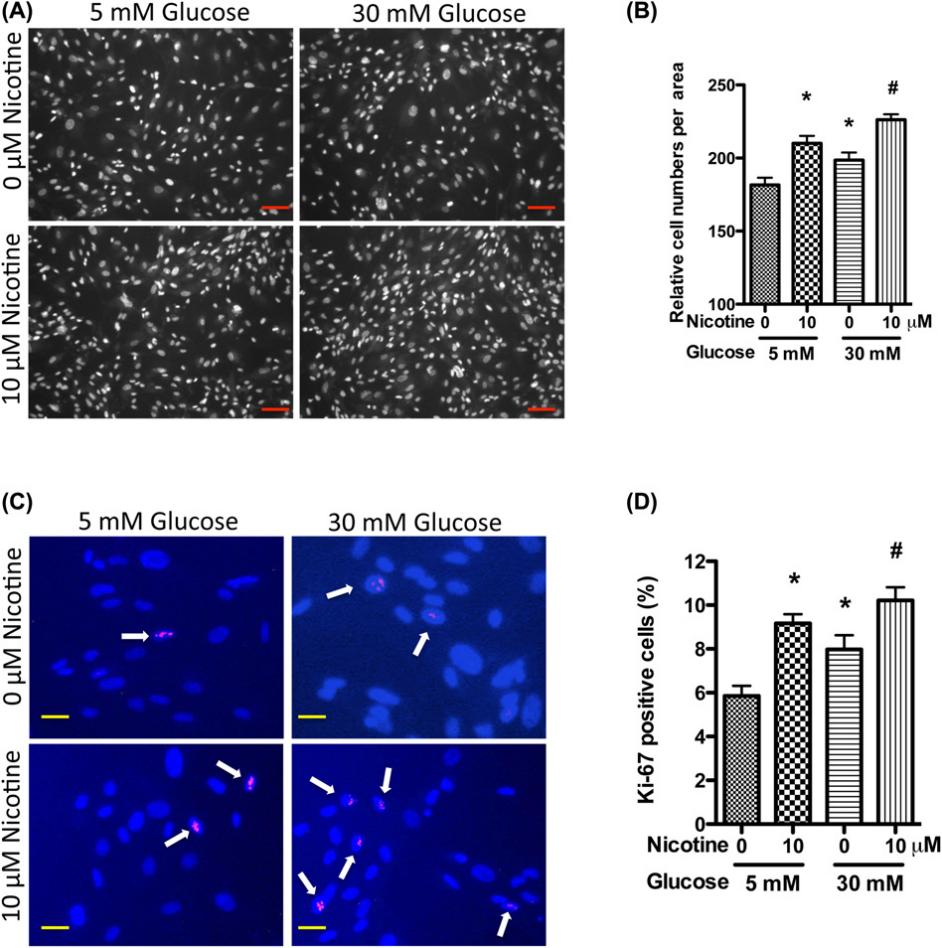
Nicotine increases mesangial cell proliferation in high glucose milieu Human mesangial cells were cultured in serum-free medium with 5 mM glucose for 24 h, and then were incubated in medium containing nicotine (10 μM) and high glucose (30 mM) for 72 h. Nuclei were stained with Hoechest 33342, and cell numbers were counted under microscope (**A**,**B**). Representative microphotographs are displayed to show cell growth (A). The cell numbers were calculated from three independent experiments (B). Cells were immunolabeled for Ki-67; representative microfluorographs are displayed (**C**,**D**). Ki-67 positive cells are indicated by arrows (C). Ki-67 positive cell ratios were calculated from three independent experiments (D). *P<0.05 in comparison with control (5 mM glucose, 0 μM nicotine) and ^#^*P*<0.05 in comparison with nicotine treatment (5 mM glucose, 10 μM nicotine). Scale bars = 100 and 20 μm for (A,C).

Nicotine has been demonstrated to increase mesangial cell proliferation and fibronectin production [17–19]. However, it is not clear whether high glucose has potential to exacerbate these phylogenic effects. To examine this aspect, we treated primary human mesangial cells with nicotine (10 μM) in the presence of normal (5 mM) or high glucose (30 mM), followed by proliferation assay by counting the cell numbers. High glucose (30 mM) increased the cell proliferation when compared with low glucose (5 mM); these findings are consistent with the previous reports [21,22]. Nicotine (10 μM) significantly increased the cell proliferation in the presence of normal glucose milieu (5 mM), but further exacerbated mesangial cell proliferation in high glucose milieu (Figure 1A,B).

The Ki-67 is a protein strictly associated with cell proliferation. During interphase, its antigen can be exclusively detected within the cell nucleus, whereas in mitosis most of the protein is relocated to the surface of the chromosomes [24]. It is present during all active phases of the cell cycle (G_1_, S, G_2_, and mitosis), but is absent from resting (quiescent) cells (G_0_) [25]. Cellular content of Ki-67 protein markedly increases during cell progression through S-phase of the cell cycle [26]. Because of these properties, Ki-67 is regarded as a cellular marker for proliferation. To confirm the effect of nicotine and high glucose on mesangial cell proliferation, we performed immunofluorescent studies to examine the changes in Ki-67 positive cell ratios. Results of these studies showed that nicotine increased the Ki-67 positive cell ratio, which was further pronounced in the presence of high glucose (Figure 1C,D).

To further confirm these findings, mesangial cells were pre-treated with high glucose for 48 h, and then treated with nicotine. Results showed that nicotine exacerbated cell proliferation in high glucose milieu when compared with low glucose milieu (data not shown). Combined together, these data indicate that high glucose enhances nicotine-induced renal mesangial cell proliferation. This is the first report to show the synergistic effect of nicotine and high glucose on the proliferation of mesangial cells.

### Nicotine enhances mesangial cell fibronectin production in high glucose milieu

**Figure 2:**
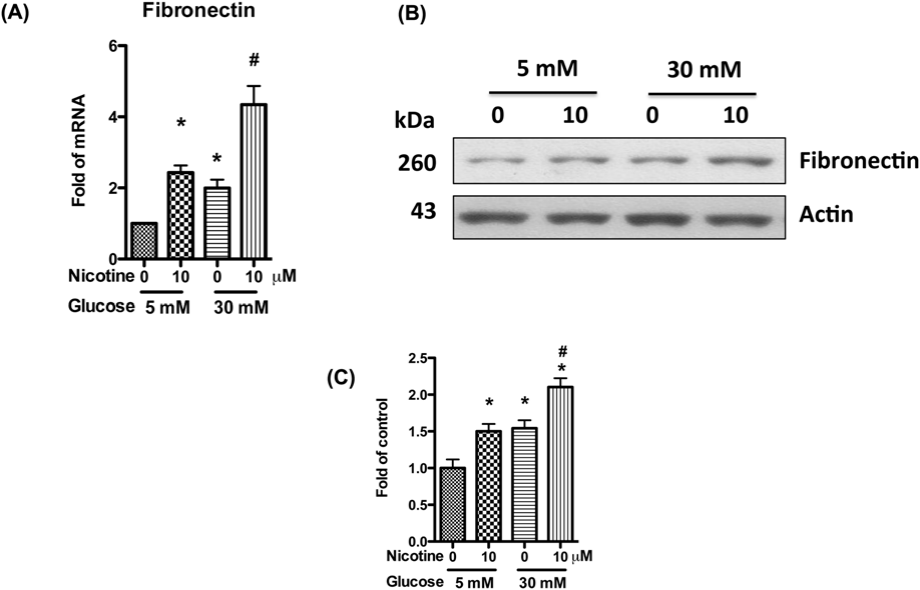
Nicotine increases mesangial cell fibronectin production in high glucose milieu Human mesangial cells were cultured in serum-free medium with 5 mM glucose for 24 h, and incubated in medium containing nicotine (10 μM) and high glucose (30 mM) for 72 h. (**A**) RNAs were extracted, and real-time PCR was performed to detect the expression of fibronectin mRNA. (**B**) Cell lysates were collected for Western blotting to detect fibronectin protein. Representative gels are shown. (**C**) The protein bands were scanned and the acquired images were analyzed using the public domain NIH image program for data quantitation. Expression of fibronectin was normalized to β-actin. Data are presented as fold of control expression. *P<0.05 in comparison with control (5 mM glucose, 0 μM nicotine), and ^#^*P*<0.05 in comparison with nicotine treatment (5 mM glucose, 10 μM nicotine).

DN is characterized by vast areas of mesangial expansion as a consequence of the accumulation of extracellular matrix. Fibronectin is a major component of extracellular matrix and accompanied with glomerular sclerosis. To examine the effect of nicotine on fibronectin expression in high glucose milieu, we treated human mesangial cells with high glucose and nicotine for 24 h, and then collected RNA for real-time PCR. Both high glucose and nicotine alone could increase the expression of fibronectin, while both glucose and nicotine combined together further increased its expression (Figure 2A).

To determine translation of fibronectin protein, Western blotting analysis was carried out with the cell lysates obtained from the abovementioned protocol. Both high glucose and nicotine alone increased the protein expression of fibronectin; however, nicotine further exacerbated high glucose-mediated mesangial cell expression of fibronectin (Figure 2B,C). Combined together, these data indicate that high glucose enhances nicotine-induced mesangial cell proliferation and fibronectin production.

### Nicotine activates Wnt/β-catenin signaling pathways in mesangial cell

**Figure 3:**
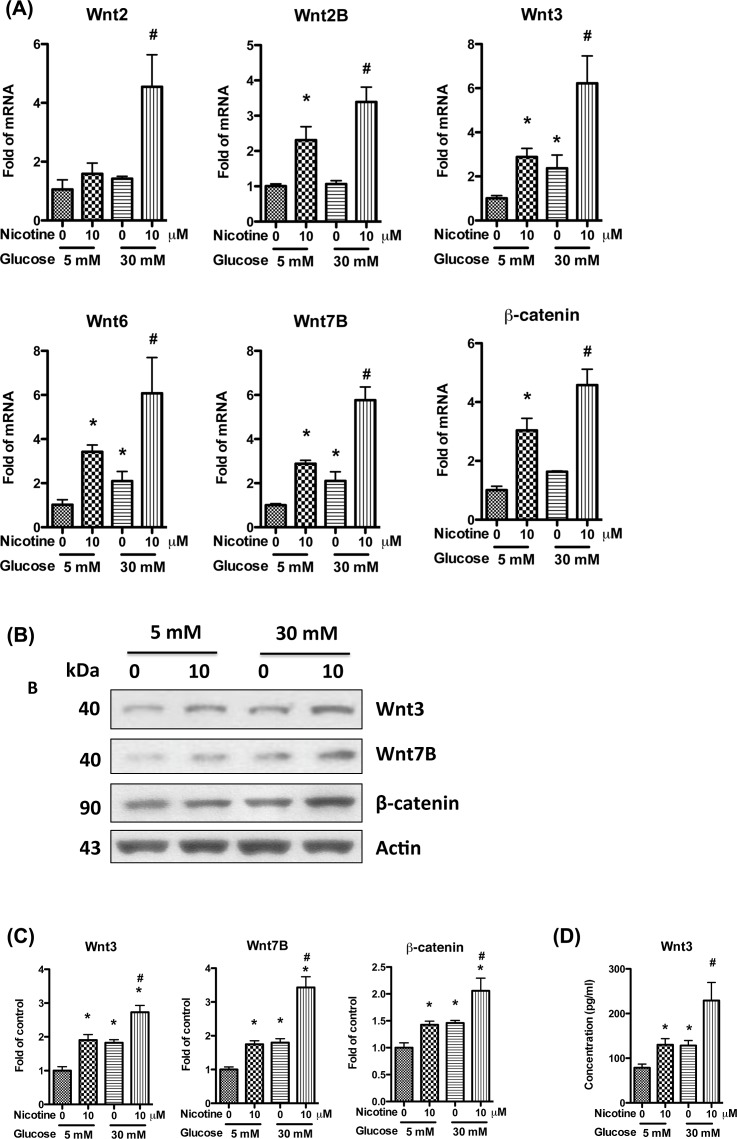
Nicotine activates mesangial cell Wnt/β-catenin pathway in high glucose milieu Human mesangial cells were cultured in serum-free medium with 5 mM glucose for 24 h, and then were stimulated for 24 h with nicotine (10 μM) and high glucose (30 mM). (**A**) RNAs were extracted and real-time PCR was performed to detect the expression of *fibronectin* mRNA. (**B**) Cell lysates were collected for Western blotting to detect the proteins of Wnt3, Wnt7B, and β-catenin. Representative gels are shown. (**C**) The protein bands were scanned and the acquired images were analyzed using the public domain NIH image program for data quantitation. Expression of Wnt3, Wnt7B, and β-catenin was normalized to β-actin. Data are presented as fold of control expression. (**D**) The mediums were collected for ELISA to determine the secretion of Wnt3. *P<0.05 in comparison with control (5 mM glucose, 0 μM nicotine), and ^#^*P*<0.05 in comparison with nicotine treatment (5 mM glucose, 10 μM nicotine).

Canonical Wnt/β-catenin signaling pathway plays an important role in the development and progression of renal fibrosis, and has been demonstrated to be a new target for the therapeutic strategy to prevent and/or slow down the progression of renal fibrosis [27–29]. In canonical Wnt/β-catenin signaling pathway, extracellular ligands (Wnts) bind to Frizzled (FZD) receptors and low-density lipoprotein receptor-related protein 5/6 (LRP 5/6) and disheveled (DSH), contributing to accumulation of cytosolic β-catenin and its import into the nucleus to act as a transcriptional coactivator of transcription factors of the TCF/LEF family. In the absence of Wnts, β-catenin will be degraded in the cytoplasm. Wnt/β-catenin signaling pathway has been demonstrated to be activated by nicotine or high glucose alone in several cell types [30–33], however, their role in mesangial cells has not been reported yet. We hypothesized that the combination of nicotine and high glucose will enhance the activation of Wnt/β-catenin signaling pathway, and their combined effect will further increase mesangial cell proliferation and fibronectin production. To validate this hypothesis, we treated human mesangial cells with high glucose (30 mM) and nicotine for 24 h, and then collected RNA for real-time PCR. Results showed that both glucose and nicotine alone could increase the expression of Wnts (2, 2A, 2B, 3, 6, and 7B), and β-catenin, while both glucose and nicotine combined together, they further increased the expression of these genes (Figure 3A).

We also collected the cell lysates and performed Western blotting. Results showed that the protein of Wnt3, Wnt7B, and β-catenin were increased by glucose and nicotine, and their combined use further increased the expression (Figure 3B,C).

To measure the Wnts in the medium of mesangial cell, we selected Wnt3 as a representative and performed ELISA. Results showed that either glucose or nicotine alone could increase the secretion of Wnt3, and their combination increased it further, which is similar with the results of Real-time PCR and Western blotting (Figure 3D). Combined together, these results validate our hypothesis that nicotine and high glucose have additive effects on the activation of Wnt/β-catenin signaling pathway in mesangial cell.

### Blocking Wnt/β-catenin signaling attenuates nicotine-induced mesangial cell proliferation and fibronectin production

**Figure 4:**
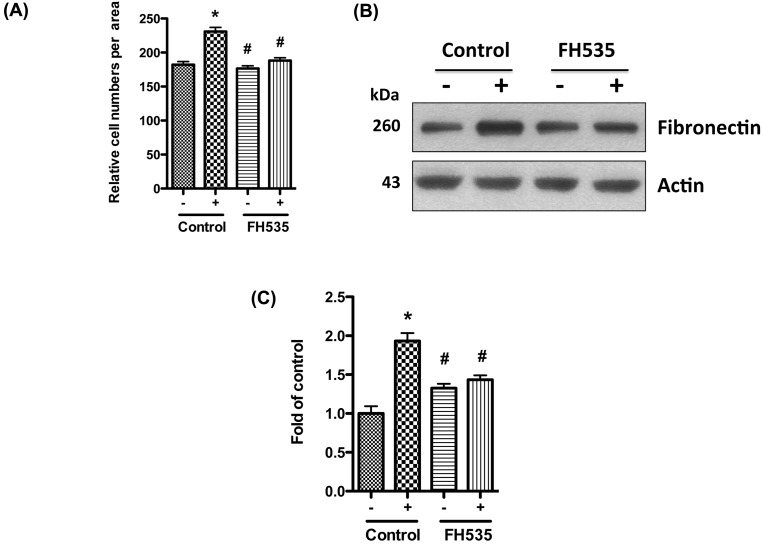
Blocking β-catenin attenuates nicotine and high glucose induced proliferation and fibronectin production Human mesangial cells were cultured in serum-free medium with 5 mM glucose for 24 h. After addition of FH535 (500 nM) for 1 h, the cells were stimulated for 72 h with nicotine (10 μM) and high glucose (30 mM). (**A**) Nuclei were stained with Hoechest 33342, and cell numbers were counted under microscope. (**B**) Cell lysates were collected for Western blotting to detect fibronectin protein. Representative gels are shown. (**C**) The protein bands were scanned and the acquired images were analyzed using the public domain NIH image program for data quantitation. Expression of fibronectin was normalized to β-actin. Data are presented as fold of control expression. *P<0.05 in comparison with control (5 mM glucose, 0 μM nicotine, no FH535), and ^#^*P*<0.05 in comparison with nicotine treatment (30 mM glucose, 10 μM nicotine, no FH535). –, 0 μM nicotine and 5 mM glucose; +, 10 μM nicotine and 30 mM glucose.

To establish a causal relationship between the activation of Wnt/β-catenin signaling and the proliferation of mesangial cell, we evaluated the effect of the blockade of the signaling pathway on mesangial cell proliferation. Since β-catenin is the key effector molecule in Wnt/β-catenin signaling pathway, we selected it as the target. We pre-treated human mesangial cells with β-catenin specific inhibitor FH535 in low glucose medium (5 mM), and then treated them with nicotine (10 μM) and high glucose (30 mM). After 3 days, we counted the cell numbers under a light microscope. Our results showed that nicotine and high glucose significantly increased the cell numbers when compared with control, while addition of FH535 abolished the mitogenic effect of nicotine and high glucose (Figure 4A). We also collected the cell lysates and performed Western blotting to detect the expression of fibronectin. FH535 significantly decreased nicotine and high glucose induced fibronectin expression (Figure 4B,C).

To further confirm this observation, we knocked down β-catenin by using siRNA, and then treated the mesangial cells with nicotine and high glucose for 3 days. Our results showed that knocking down β-catenin also decreased nicotine-induced mesangial cell proliferation and fibronectin production (data not shown). Combined together, we confirmed that blockade of β-catenin attenuates nicotine induced mesangial cell proliferation and fibronectin production in high glucose milieu.

In the present study, we investigated the effect of nicotine and high glucose on mesangial cell. We found that nicotine could exacerbate high glucose induced mesangial cell proliferation and fibronectin production. Both nicotine and high glucose activated Wnt/β-catenin signaling pathway in mesangial cell, and their combined use further activated this pathway. Blocking Wnt/β-catenin pathway with β-catenin specific inhibitor or siRNA significantly attenuated nicotine and high glucose induced mesangial cell proliferation and fibronectin production. These results suggest that nicotine increases mesangial cell proliferation and fibronectin production in high glucose milieu through Wnt/β-catenin pathway. Our study highlights new potential targets for the prevention and therapy of DN.

## Abbreviations

DN: diabetic nephropathy
nAChR: nicotinic ACh receptor
